# Mapping tissue inhomogeneity in acute myocarditis: a novel analytical approach to quantitative myocardial edema imaging by T2-mapping

**DOI:** 10.1186/s12968-015-0217-y

**Published:** 2015-12-23

**Authors:** Bettina Baeßler, Frank Schaarschmidt, Anastasia Dick, Christian Stehning, Bernhard Schnackenburg, Guido Michels, David Maintz, Alexander C. Bunck

**Affiliations:** Department of Radiology, University Hospital of Cologne, Kerpener Str. 62, D-50937 Cologne, Germany; Institute of Biostatistics, Faculty of Natural Sciences, Leibniz Universität Hannover, Hannover, Germany; Philips Research, Hamburg, Germany; Philips, Healthcare Germany, Hamburg, Germany; Department III of Internal Medicine, Heart Centre, University Hospital of Cologne, Cologne, Germany

**Keywords:** Cardiovascular magnetic resonance, Tissue characterization, Acute myocarditis, T2-mapping, Myocardial edema

## Abstract

**Background:**

The purpose of the present study was to investigate the diagnostic value of T2-mapping in acute myocarditis (ACM) and to define cut-off values for edema detection.

**Methods:**

Cardiovascular magnetic resonance (CMR) data of 31 patients with ACM were retrospectively analyzed. 30 healthy volunteers (HV) served as a control. Additionally to the routine CMR protocol, T2-mapping data were acquired at 1.5 T using a breathhold Gradient-Spin-Echo T2-mapping sequence in six short axis slices. T2-maps were segmented according to the 16-segments AHA-model and segmental T2 values as well as the segmental pixel-standard deviation (SD) were analyzed.

**Results:**

Mean differences of global myocardial T2 or pixel-SD between HV and ACM patients were only small, lying in the normal range of HV. In contrast, variation of segmental T2 values and pixel-SD was much larger in ACM patients compared to HV. In random forests and multiple logistic regression analyses, the combination of the highest segmental T2 value within each patient (maxT2) and the mean absolute deviation (MAD) of log-transformed pixel-SD (madSD) over all 16 segments within each patient proved to be the best discriminators between HV and ACM patients with an AUC of 0.85 in ROC-analysis. In classification trees, a combined cut-off of 0.22 for madSD and of 68 ms for maxT2 resulted in 83 % specificity and 81 % sensitivity for detection of ACM.

**Conclusions:**

The proposed cut-off values for maxT2 and madSD in the setting of ACM allow edema detection with high sensitivity and specificity and therefore have the potential to overcome the hurdles of T2-mapping for its integration into clinical routine.

## Background

Acute myocarditis (ACM) is one of the leading causes of sudden cardiac death in young adults and is found in up to 20 % of autopsy cases [1]. Moreover, it represents a frequent precursor of dilated cardiomyopathy (DCM) [2]. Unfortunately, clinical presentations and symptoms largely vary, as does the clinical course of disease. The diagnostic tools for detecting ACM lack diagnostic sensitivity and specificity, including the current “gold standard” endomyocardial biopsy [3]. Therefore, the diagnosis of ACM still remains a great challenge.

During the last decade, cardiovascular magnetic resonance (CMR) with its unique capability of tissue characterization has become the reference non-invasive imaging technique for the diagnosis of ACM [4, 5]. It has the potential to identify the various hallmarks of myocardial inflammation, including edema, hyperemia and fibrosis by using T2-weighted imaging, early and late gadolinium enhancement (EGE, LGE), referred to as the Lake Louise consensus criteria (LL criteria) [6].

Especially imaging myocardial edema, however, remains a great challenge as conventional qualitative T2-weighted imaging suffers from various limitations [7, 8]. Recently, T2-mapping has been suggested as a quantitative approach to edema imaging, allowing for a more sensitive detection of either diffuse or even subtle changes in myocardial T2 relaxation times [7, 8]. One of the main challenges of myocardial T2-mapping, however, is the high intra- and interindividual variability of T2 times, leading to potential difficulties in discriminating between health and disease. While T2 is an inherent tissue property, a recent study in healthy volunteers (HV) demonstrated that myocardial T2 varies significantly depending on the sequence type and field strength used [9], leading to variations of T2 times of almost 20 ms. Considering that the difference between remote and edematous myocardium has been reported to be in the range of 10–20 ms [7, 10], we hypothesized that dedicated segmental reference values are needed for a future diagnostic decision making.

The present study therefore aims at establishing a cut-off value of myocardial T2 relaxation times for the accurate detection of myocardial edema in the setting of ACM, thereby evaluating the usefulness of the proposed segmental reference values for the Gradient Spin Echo (GraSE) T2-mapping sequence at 1.5 T [9]. In addition, we previously hypothesized that ACM will result in an increased inhomogeneity of T2 times due to its often focal disease manifestation [9, 11]. Therefore, we intend to analyze the diagnostic value of additional parameters that reflect increased variability of T2 times between and within the myocardial segments.

## Methods

### Study population

After obtaining approval by the Institutional Review Board of the University Hospital of Cologne, a retrospective study was performed. Data sets of 31 patients who had been consecutively referred to our department for CMR from 2012 to 2014 after clinical diagnosis of ACM were retrospectively analyzed in a systematic fashion. The clinical diagnosis of ACM was based upon the current recommendations given by the position statement of the European Society of Cardiology Working Group on Myocardial and Pericardial Diseases [12] (Table 1). CMR diagnosis of ACM was based upon the presence of at least two out of three LL Criteria [6], i.e. visually detected myocardial edema on T2-weighted images, early gadolinium enhancement or visually detected LGE.

**Table 1:** Classification of ACM patients (*n* = 31) according to the current recommendations [12]

	ACM (n)
Clinical presentation	
Acute chest pain	23
New-onset (days up to 3 months) or worsening of: dyspnoea at rest or exercise/fatigue, with or without left and/or right heart failure signs	16
Palpitations/arrhythmia symptoms/syncope/aborted sudden cardiac death	18
Cardiogenic shock	1
Diagnostic criteria	
ECG/Holter/stress test features	31
Elevated TnT/TnI	26
Functional and structural abnormalities on cardiac imaging (echo/angio/CMR)	31
Tissue characterization by CMR	
Visual edema (T2-weighted imaging)	23
T2-Ratio ≥1.9 [6, 22]	25
Early Enhancement Ratio (EGEr) >4	17
Visual LGE of non ischemic pattern	30
Edema + EGEr positive	1
Edema + LGE positive	9
EGEr + LGE positive	6
3 out of 3 criteria positive	15
Exclusion of CAD by coronary angiography	31

CMR data from 30 HV served as control. The status “healthy” was based on: i) uneventful medical history, ii) absence of any symptoms indicating cardiovascular dysfunction iii) normal cardiac dimensions and function proven by cine CMR, and iv) no history of inflammatory disease including common cold virus in the last 4 weeks before the examination [13]. We discouraged alcohol intake 24 h before the scans to avoid inflammatory reaction [14]. For each volunteer written informed consent was obtained prior to the study after approval by the local ethics committee. Characteristics of patients and HV are shown in Table 2.

**Table 2:** Characteristics of patients and healthy volunteers

Parameter	Healthy volunteers	ACM patients
Number	30	31
Females/Males	16/14	6/25
Age [years]	36 ± 13	40 ± 15
Age group 20–34 years	17	15
Age group 35–49 years	9	9
Age group 50–75 years	4	7
Height [cm]	176 ± 9	175 ± 9
Weight [kg]	73 ± 14	79 ± 17
Initial TnT [μg/l]	n.a.	0.56 ± 0.68
Initial NT-proBNP [pg/ml]	n.a.	3201 ± 6389
Initial CK [U/l]	n.a.	514 ± 609
LV enddiastolic volume/BSA [ml/m^2^]	80 ± 14	86 ± 15
LV endsystolic volume/BSA [ml/m^2^]	31 ± 8	42 ± 16
LV ejection fraction [%]	62 ± 5	55 ± 13
LV ED wall mass/BSA [g/m^2^] (without papillary muscles)	44 ± 10	66 ± 12
T2-Ratio	1.7 ± 0.4	2.2 ± 0.4
T2-Ratio >1.9	10	25
Global myocardial T2 [ms]	58.7 ± 4.2	62.1 ± 7.2
Range of global T2 values [ms]	47.7–66.4	49.3–76.9
Global pixel-SD [ms]	7.7 ± 1.9	8.5 ± 2.4
Range of pixel-SD values [ms]	5.5–13.9	5.1–16.5

### CMR

CMR was performed on a 1.5 T MR system (Achieva 1.5 T, Philips Healthcare, Best, The Netherlands) using a standard five-element cardiac phased array coil and a 4-lead vectorcardiogram. A balanced steady-state free precession (b-SSFP) sequence in breath-hold technique and with retrospective ECG-triggering was acquired for functional analysis. Imaging parameters were chosen as follows: repetition time (TR) 28 ms, echo time (TE) 1.4 ms, flip angle (FA) 60°, field of view (FOV) 343 × 380 mm^2^, matrix 256 × 256, slice thickness 8 mm, 50 cardiac phases.

In all patients and HV the MR protocol included three horizontal long axes and a stack of short axes (SAX) covering the left ventricle (LV) to assess wall motion and allow for cardiac chamber quantification. Volumetry was performed on a standard post-processing platform (Extended MR WorkSpace, Version 2.6.3.4, Philips Healthcare, Best, The Netherlands).

Edema-sensitive black blood T2-weighted images with fat saturation (T2 BB) in six SAX slices were used to visualize inflammatory changes in the myocardium of HV and ACM patients [15]. In order to detect myocardial hyperemia, myocardial early gadolinium enhancement was assessed in ACM patients using fast spin- echo T1-weighted images during the first minutes after 0.1 mmol/kg Gd-DOTA (Dotarem; Guerbet, Villepinte, France) contrast administration as previously described [16]. For the additional detection of myocardial fibrosis and scarring, LGE imaging was performed in ACM patients 15 min after 0.2 mmol/kg Gd-DOTA (Dotarem; Guerbet, Villepinte, France) contrast administration using an inversion-recovery gradient-echo sequence in the horizontal long axes and SAX as previously described [17].

#### T2-mapping

In order to cover most of the left ventricle and not to miss focal edema, T2-mapping data were acquired in six SAX slices evenly distributed across the LV using an ECG-triggered, breathhold Gradient Spin Echo technique (GraSE) [11, 18]. Image parameters were chosen as follows: spatial resolution: 2 × 2 × 10 mm^3^, TR = 1 heartbeat, nine echoes (TE_1_ = 15 ms, delta TE = 7.7 ms), FA 90°, parallel imaging (SENSE = 2), EPI factor = 7, BlackBlood-prepulse. A pixel-wise myocardial T2-map was generated using a mono-exponential fit [7, 8, 19] on the magnitude data using a maximum likelihood estimator (MLE), where a Rician noise distribution was assumed.

### CMR image analysis

#### Lake Louise criteria

Image analysis of Lake Louise Criteria was performed using a standard post-processing platform (Extended MR WorkSpace, Version 2.6.3.4, Philips Healthcare, Best, The Netherlands). The myocardium was divided into 16 segments according to the AHA 16-segment model [20, 21]. Every segment was visually evaluated for presence of myocardial edema on T2 BB imaging. Moreover, T2-ratio was calculated as previously described [6, 22]. For calculation of the Early Gadolinium Enhancement Ratio (EGEr), standardized myocardial and skeletal muscle regions of interest (ROIs) were drawn on one axial slice before and after contrast administration. EGEr was calculated as previously described [23]. Every segment was visually assessed on LGE images and considered suspicious for myocarditis in cases of focal signal intensity alterations with a pattern typical for myocarditis [6].

#### T2-mapping

T2-map reconstruction and image analysis was done with OsiriX viewer for Mac OS X (version 5.8.5, Pixmeo, Bernex, Switzerland). T2-maps were calculated with a dedicated plug-in written for the OsiriX software. An endocardial and epicardial contour was drawn in one original image. The trabeculated layer and the epicardial border were left out. In doubtful cases, SSFP cine images were consulted. The contours were copied to the other images and aligned in each source image to correct for respiration-induced rigid body motion. These final contours were copied to the map and corrected when necessary. The myocardial ROI was automatically segmented according to the AHA 16-segment model [20, 21] and T2 values were calculated for each segment. While averaging all pixels within one myocardial segment for segmental T2 calculation, the corresponding standard deviation was recorded and assigned to an additional parameter we refer to as “pixel-SD”.

### Statistical analysis

Statistical analysis was performed in R 3.1.2 [24], using the packages ggplot2 [25] for graphical visualization, pastecs [26] for descriptive statistics, DAAG [27] for cross-validation, randomForest [28] for random forests, rpart [29] for fitting single classification trees [30], and ROCR [31] for ROC analyses.

All continuous data are given as mean ± standard deviation. A *p*-value of < .05 was regarded as statistically significant. Testing for significant differences between patients and HV was performed using Welch independent *T*-test and Wilcoxon sum rank test. Classification models were built using multiple as well as multinominal logistic regression analyses. We further used sets of classification trees [30] with an internal weighting by cross validation (random forests [28]), to identify variables that are empirically important for classification into ACM and HV with a low misclassification rate. When assessing variable importance using random forests, the Gini-Index was used as a measure for impurity of classification [28]. To visualize the cut-offs that are best for the current data, single classification trees (also known as recursive partitioning) were fitted, again using the Gini-index as a measure for impurity [30]. The diagnostic accuracy of optimal predictive parameters was evaluated using receiver operating curve (ROC) analyses.

## Results

### Basic demographic data

Data on clinical presentation and diagnostic criteria according to the current recommendations [12] are given in Table 1. Demographic characteristics of HV and ACM patients as well as LV volumetric data, global T2 and pixel-SD values are presented in Table 2.

### T2-mapping in CMR-proven ACM

**Fig. 1:**
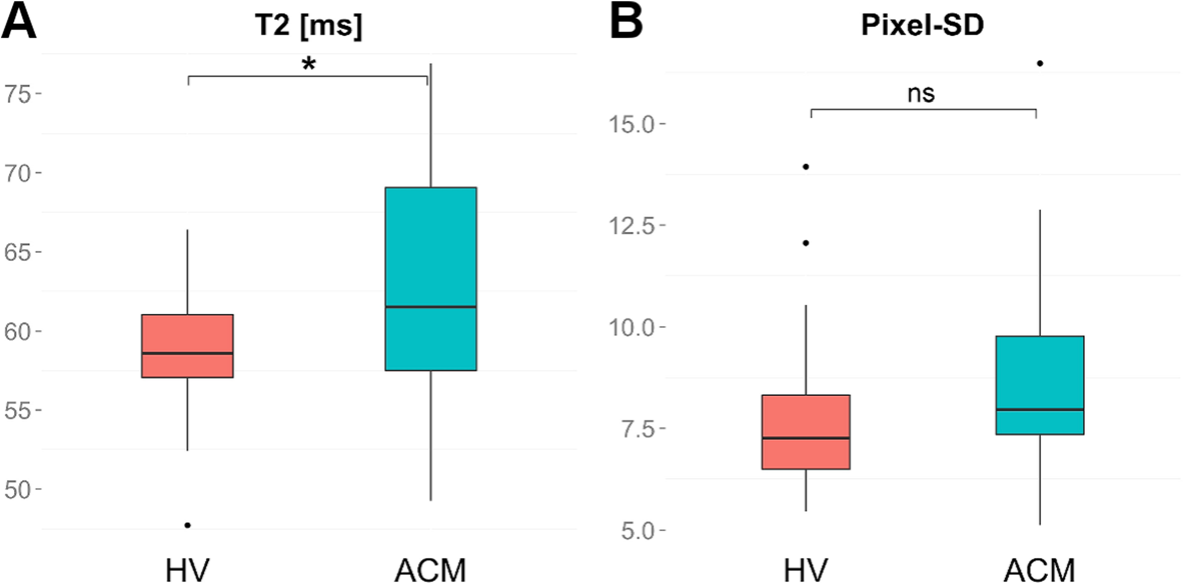
Box-Whisker plots representing the differences of global myocardial T2 (**a**) and pixel-SD (**b**) between HV and ACM patients. The centreline in each box represents the median, whereas the lower and upper limits of each box represent the 25^th^ and 75^th^ percentiles, respectively. Whiskers extend to the most extreme observations within 25^th^ and 75^th^ percentiles ± 1.5*IQR. Observations outside these whiskers are shown as dots. *** IQR - inter-quartile-range, ACM - acute myocarditis, HV - healthy volunteers

Global myocardial T2 times (i.e. mean segmental T2 values averaged over all 16 segments) in ACM patients were significantly higher compared to HV (Table 3, Fig. 1a), although this small difference yielded an only small to medium effect size (Table 3) and did not exceed the normal range of T2 values in HV (Table 2). Global myocardial pixel-SD showed a large overlap between ACM patients and HV (Table 3, Fig. 1b).

**Table 3:** Means ± standard deviation, statistical significances and effect sizes of myocardial T2, pixel-SD and statistically derived parameters

Parameter	Healthy volunteers	ACM patients	*p*-value	Effect size r
Global myocardial T2 [ms]	58.7 ± 4.2	62.1 ± 7.2	.026	.31
Global pixel-SD [ms]	7.7 ± 1.9	8.5 ± 2.4	.166	.18
maxT2 [ms]	69.6 ± 8.5	80.5 ± 16.8	.003	.43
madT2	0.07 ± 0.03	0.09 ± 0.02	.060	.26
maxSD [ms]	12.5 ± 4.2	19.0 ± 8.6	.001	.50
madSD	0.22 ± 0.05	0.29 ± 0.06	.001	.56

In a previous study in HV [9], our group established layer-specific myocardial reference T2 values for the GraSE sequence at 1.5 T (i.e. 56.7 ± 4.1 ms for the basal, 58.1 ± 4.2 ms for the midventricular, and 62.2 ± 5.2 ms for the apical layer). Applying these cut-off values plus a two-fold standard deviation on the present patient cohort resulted in a classification of ACM patients with 26 % false-negative and 43 % false-positive results. Increasing reference values to a cut-off plus a three-fold standard deviation resulted in a classification with 37 % false-negative and 23 % false-positive results.

**Fig. 2:**
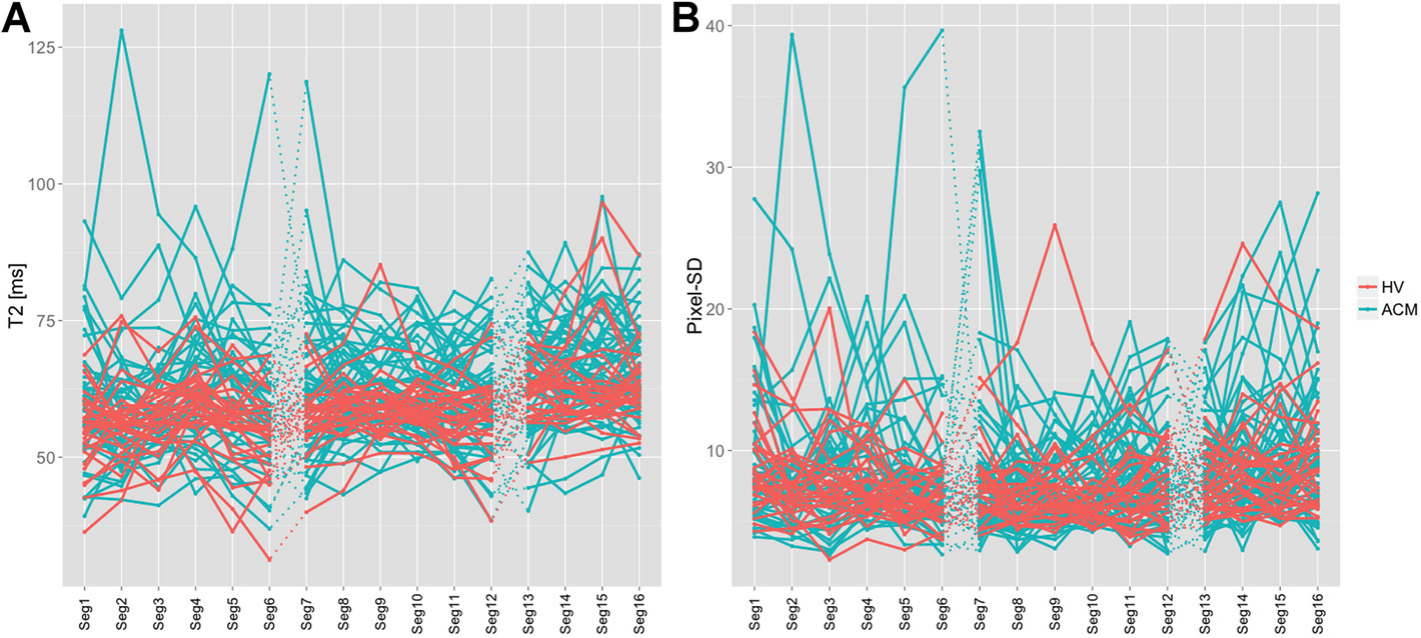
Individual variation of segmental values for myocardial T2 times (**a**) and pixel-SD (**b**) over all 16 segments, where each line represents an individual subject, thereby comparing HV (*red*) and ACM patients (*blue*). ACM - acute myocarditis, HV - healthy volunteers

Graphical visualization of segmental T2 values and pixel-SD (Fig. 2) underlined that means of both parameters showed no large differences. In contrast, the graphics revealed that variation of T2 values and pixel-SD was much larger in ACM patients compared to HV. Taking into account the often focal nature of ACM, these observations led to the assumption that a cut-off neither for average nor for layer-specific or segmental myocardial T2 values based on reference T2-maps would be able to reliably differentiate between health and focal disease.

### Calculation of statistically derived parameters of T2 and pixel-SD

**Fig. 3:**
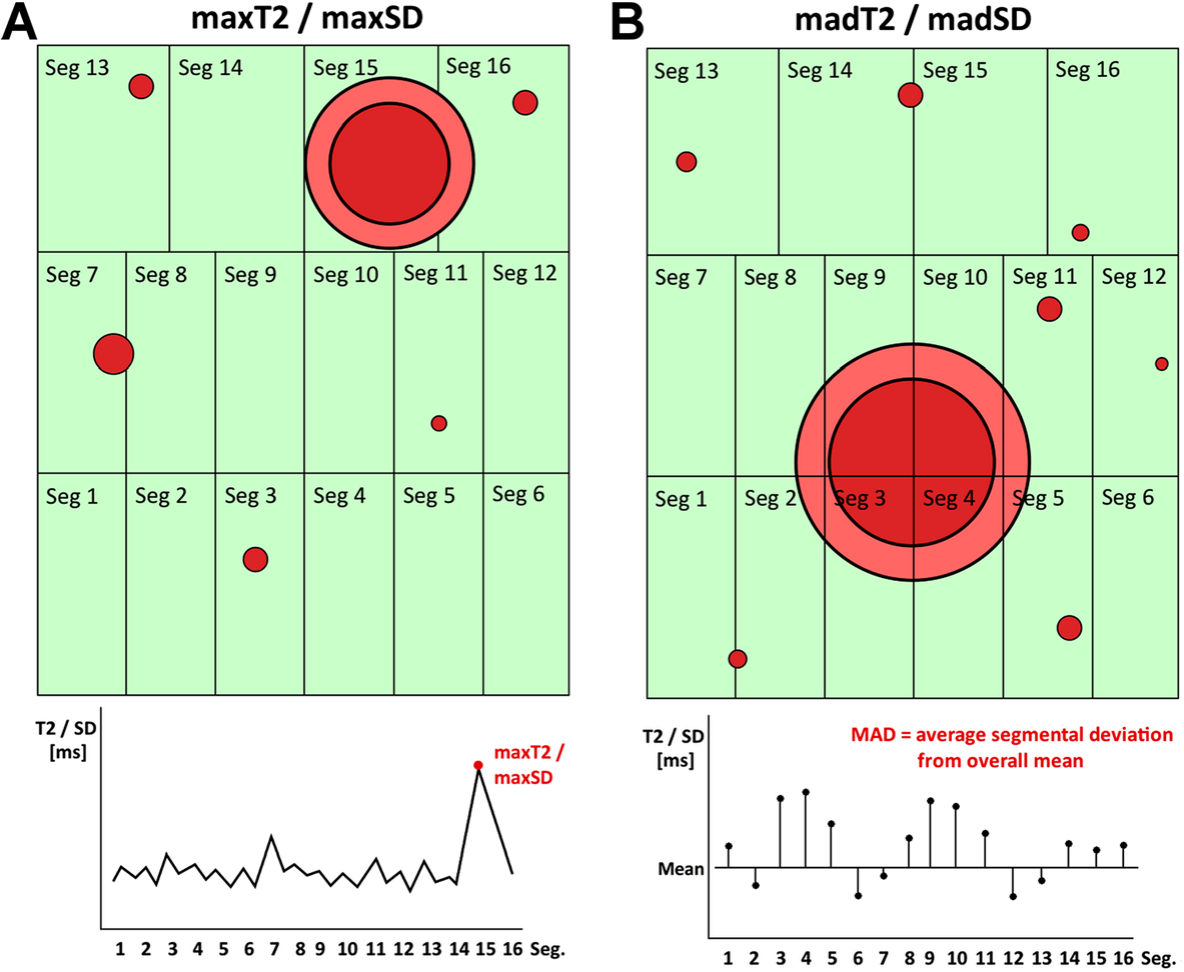
Graphical illustration of the novel concepts of maxT2/maxSD (**a**) and madT2/madSD (**b**). Each box represents one segment (1–16) of one individual. *Red dots* represent focal spots of myocardial inflammation. The graphs at the bottom illustrate the corresponding individual T2/Pixel-SD values, aufgetragen over all 16 segments in order to define maxT2/maxSD (**a**) as well as madT2/madSD (**b**). In **b**, *black horizontal lines* indicate the mean across segments. *Vertical black lines* for madT2/madSD illustrate the deviations from mean that are averaged to obtain the MAD. MAD - mean absolute deviation. Seg. - segment

In order to find parameters that might be able to detect edema more reliably, we defined several parameters derived from segmental T2 and log-transformed pixel-SD values (natural logarithm), aiming at reflecting the main aspects of focal pathologies (Fig. 3): a) the maximum segmental T2 and pixel-SD value (maxT2, maxSD; defined as the one segment of all 16 segments exhibiting the highest segmental T2 or the highest pixel-SD value (Fig. 3a), respectively) as sensitive parameters for single or multiple edematous segments (and also for the case of diffuse myocardial edema) and especially for a situation where only a single segment is (partially) involved in focal edema, and b) the mean absolute deviation (MAD) of segmental T2 and log_e_-transformed pixel-SD (madT2, madSD) as sensitive parameters for several adjacent or non-adjacent segments only partially involved in focal myocardial edema (Fig. 3b). The MAD is a measure of the variability between segments (comparable to the variance between segments), which becomes high if some segments are affected while others are not. Because MAD measures how far (in average) the values in the single segments differ from the overall myocardial mean, it is not affected by the high between subject variability that had been shown in earlier studies [9]. Moreover, MAD is a robust measure of variation not so much affected by single outlying observations as would be the variance or standard deviation.

To avoid confusion due to the different use of the abbreviation MAD, the definition of madSD used herein is given in the following: denote with *x*_*j*_ the observed pixel-SD in the *k* segments within a given subject, with index *j = 1,…,k*. These values have been transformed using the natural logarithm, and the average has been computed on this transformed scale.

$$ {y}_j={ \log}_e\left({x}_j\right), \kern0.24em \overline{y}=\frac{{\displaystyle \sum_{j=1}^k{y}_j}}{k} $$

Then, the mean (average) absolute deviation of the segments’ transformed values from the average on the transformed scale is computed, without any additional re-scaling:

$$ madSD=\frac{{\displaystyle \sum_{j=1}^k\left|{y}_j-\overline{y}\right|}}{k} $$

In the same way, madT2 has been computed as the average absolute difference of segmental T2 values from the overall mean of T2, but without log-transformation.

These parameters, as well as the subject-specific means across all segments for T2 and pixel-SD were included in further statistical analyses.

### Data distribution of maxT2/maxSD and madT2/madSD and model building

**Fig. 4:**
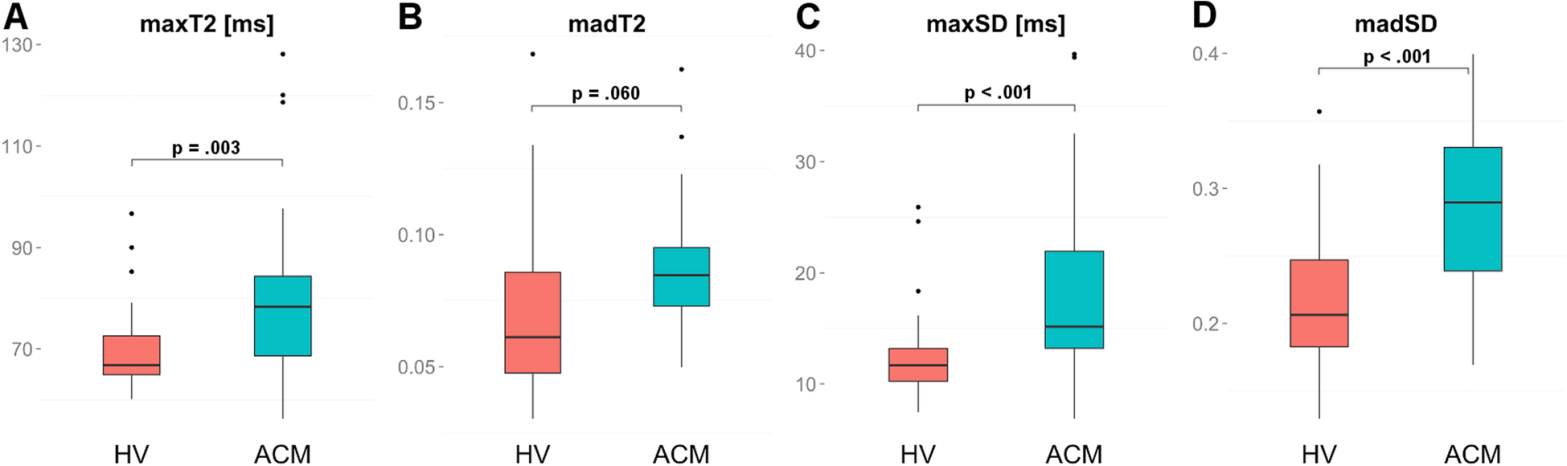
Box-Whisker plots representing the differences of maxT2 (**a**), madT2 (**b**), maxSD (**c**), and madSD (**d**) between HV and ACM patients. The centreline in each box represents the median, whereas the lower and upper limits of each box represent the 25^th^ and 75^th^ percentiles, respectively. Whiskers extend to the most extreme observations within 25^th^ and 75^th^ percentiles ± 1.5*IQR. Observations outside these whiskers are shown as dots. *** IQR - inter-quartile-range, ACM - acute myocarditis, HV - healthy volunteers

There were significant differences of maxT2, maxSD and madSD between HV and ACM patients (Table 3, Fig. 4). MadT2 only showed a non-significant trend towards slightly higher values in ACM patients compared to HV (Table 3, Fig. 4).

**Fig. 5:**
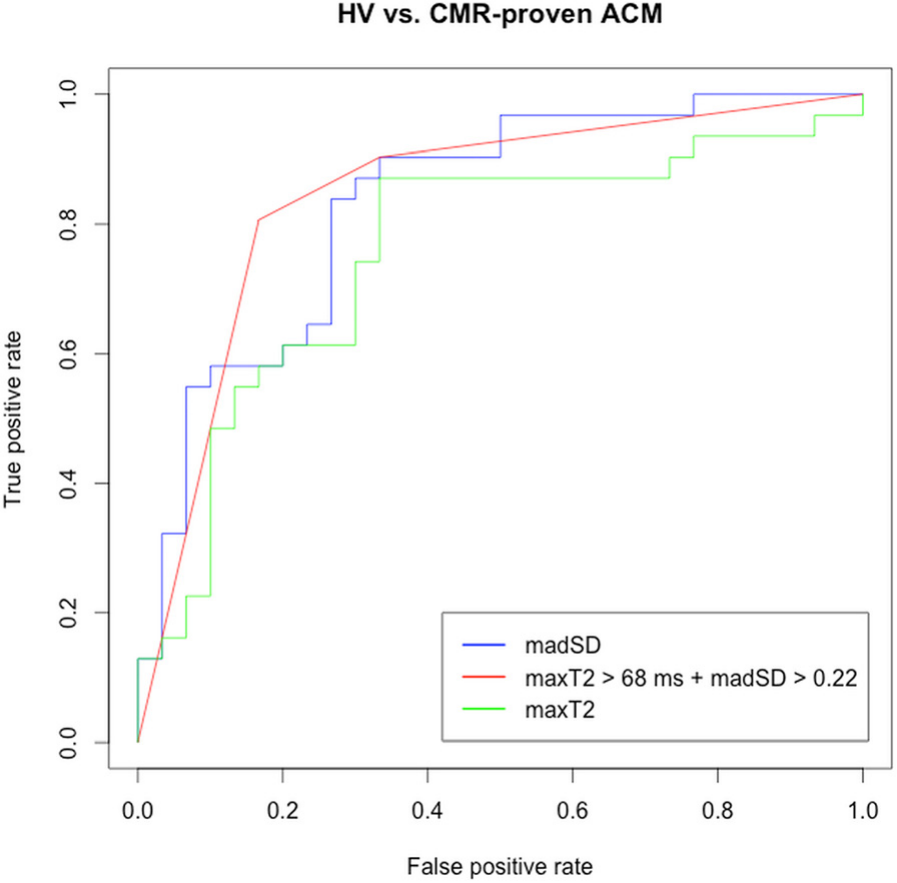
ROC-Analysis for differentiating CMR-proven ACM patients from HV. ACM - acute myocarditis, HV - healthy volunteers

**Fig. 6:**
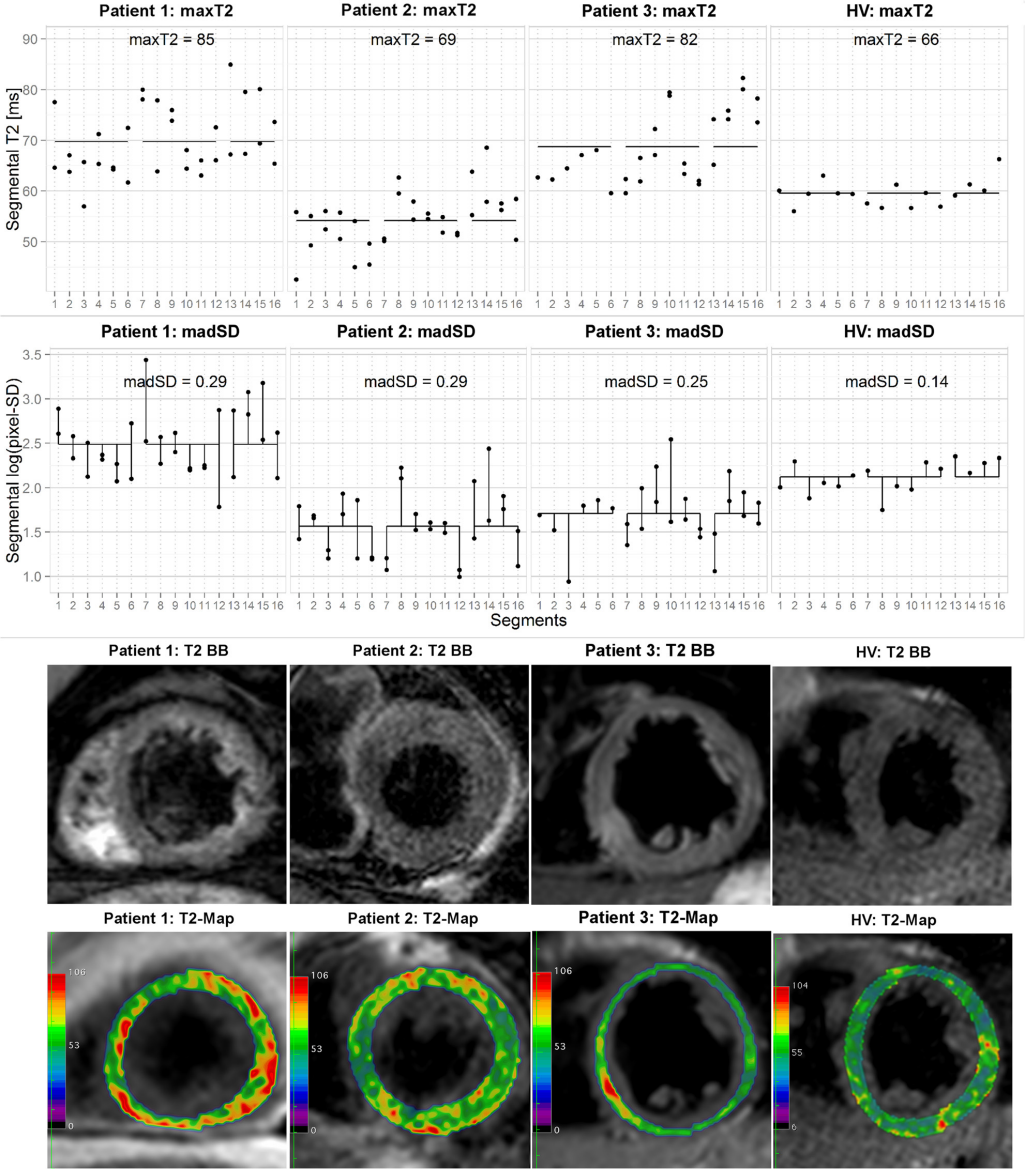
Segmental T2 (first row), log-transformed pixel-SD (second row), T2 black-blood images of one short axis slice, and corresponding T2-maps for 4 selected subjects. *Black horizontal lines* indicate the mean across segments. *Vertical black lines* for madSD illustrate the deviations from mean that are averaged to obtain madSD

In exploratory random forests, madSD, madT2 and maxT2 proved to be the best classifying parameters according to the Gini index. In multiple logistic regression analyses, madSD and maxT2 proved to be the best independent predictors of CMR-proven ACM (Table 4) according to the Akaike Information Criterion (AIC). Accordingly, if madSD increased by 0.1, the odds (p(ACM)/p(HV)) increased by factor 7.64; if maxT2 increased by 1, the odds (p(ACM)/p(HV)) increased by factor 1.03. Due to collinearity between maxT2 and madSD, however, maxT2 was not significant when included in a model that also contained madSD as a predictor. In a 10-fold cross-validation, this model yielded an internal estimate of accuracy of 0.75 and a cross-validation estimate of accuracy of 0.72. The logistic regression as well as the corresponding random forest model showed a classification error of 27 % false-positive and 26 % false-negative classifications.

**Table 4:** Multiple logistic regression model

		95 % CI for odds ratio		
	B (SE)	Lower	Odds ratio	Upper
Intercept	−7.00			
maxT2	0.03	0.97	1.03	1.10
madSD	20.34	2.30	7.64	32.71

### Definition of cut-off values

For further validation and illustration, we estimated cut-off values for both parameters, madSD and maxT2 using classification trees [30]. This led to the definition of a cut-off of 0.22 for madSD and of 68 ms for maxT2. Values below both cut-offs were more likely to represent HV, values above more likely to represent ACM. Applying both cut-offs in combination allowed a classification of ACM patients with a sensitivity of 81 % and a specificity of 83 % and showed an AUC of 0.84 in ROC-analysis (Fig. 5). In all 8 patients that visually did not reveal myocardial edema, edema could be detected using the two cut-off values. ROC-analyses for the single parameters maxT2 (67 % sensitivity, 87 % specificity) and madSD showed an AUC of 0.76 and 0.83 (83 % sensitivity, 73 % specificity), respectively (Fig. 5).

### Feasibility of the defined parameters for the diagnosis of ACM

Figure 6  shows the profiles of segmental T2 values and log-transformed segmental pixel SDs for three exemplarily selected subjects (two ACM patients and one HV) with corresponding representative T2 BB images and T2-maps. Patient 1 exhibits high average T2 (80 ms), high maxT2 (119 ms) accompanied by high pixel-SD and madSD (0.29). This subject may represent a part of the population of ACM patients that could be identified on rules either based on mean T2 or maxT2, as well as on measures of within-segment heterogeneity such as madSD. T2 BB imaging of this patient shows no visually apparent focal edema, whereas high maxT2 is already apparent visually in the anterior wall of the midventricular portion of the LV on the T2-map, what is in concordance with the segmental distribution of maxT2 and madSD.

In contrast, ACM patient two shows relatively low average T2 values (54 ms) and no extreme peak of segmental T2-values, resulting in a maxT2 of 69 ms. However, this patient exhibits high madSD values, indicating that several segments show high within segment T2 variability. This may be the result of an ACM focus that is subdivided among several segments, so that none of the affected segments shows an extraordinarily high T2 but all affected segments show a high positive deviation from the mean pixel-SD. The high intramyocardial inhomogeneity is also visible on the T2-map, whereas T2 BB imaging shows neither an unequivocal focal nor a diffuse rise of signal intensity.

Finally, the selected HV shows low average T2 (59 ms), segmental T2 values with only low deviations from the average T2 and only limited variation of within segment heterogeneity. The corresponding T2-map reflects low myocardial T2 as well as relative homogeneous myocardial T2.

### Relation of diagnostic parameters to laboratory testing

Patients with CMR-proven ACM showed a mean initial Troponin T (TnT) of 0.59 μg/l, a mean initial brain natriuretic peptide (NT-proBNP) of 3201 pg/ml and a mean initial creatine kinase (CK) of 514 U/l. Patients diagnosed with ACM according to the above defined criteria (maxT2 >68 ms + madSD >0.22, *n* = 21) exhibited higher initial TnT values compared to the remaining CMR-proven ACM patients, although this difference was not significant at the 5 % level (0.59 ± 0.71 vs. 0.48 ± 0.62, *p* = .641). Patients with pathologic T2 values according to the definition showed significantly higher initial NT-proBNP values (3650 ± 6780 vs. 279 ± 186 pg/ml, *p* = .038) as well as a non-significant trend towards higher CK values (572 ± 673 vs. 331 ± 309 U/l, *p* = .500).

## Discussion

Myocardial edema is an important pathophysiological component in many acute heart diseases such as myocarditis. T2-mapping may allow for a more sensitive and objective detection of changes in myocardial water content by means of altered T2 relaxation times [7, 8]. Its integration into clinical routine, however, is still hindered by several limitations, despite some previous attempts to define appropriate cut-off values for myocardial T2 times [7, 32–35]. In addition, several studies have presented partially contradictory results with respect to the diagnostic potential of T2-mapping in the setting of myocarditis [7, 33, 34, 36]. In the present study, we sought to address one of the main challenges of T2-mapping, i.e. the high intra- and interindividual variability of T2 times that so far have led to difficulties in discriminating between health and disease. We hereby present a potential novel diagnostic parameter, madSD that is aimed at reflecting the inhomogeneity of myocardial tissue due to inflammatory changes, as further discussed below. In addition, we present dedicated cut-off values for madSD in combination with maximal segmental T2 (maxT2), aiming at a more sensitive and specific detection of myocardial edema in the setting of ACM.

There have been several attempts and different approaches to bring T2-mapping towards clinical routine in the setting of myocarditis. Thavendiranathan et al. [7] established a cut-off of 59 ms for myocardial T2 using the T2prep sequence [8] by averaging T2 over segments rated as “involved”/“not involved” visually as well as by relying on wall motion abnormalities. Wassmuth et al. defined a similar cut-off of 60 ms for T2prep while comparing T2 of remote and edematous segments of ACM patients [35]. Butler et al. published a cut-off of 59 ms that was able to predict biopsy-proven myocarditis using a multi echo spin echo approach (MESE) by drawing a single ROI in the septum [32]. Trying to establish a single cut-off for myocardial T2 by using the rule “reference value plus 2- or 3-fold SD” in our study, however, resulted in unsatisfactory classification rates of “healthy” and “ACM” with either too many false-positive or false-negative classifications.

All these approaches have a common major limitation: averaging myocardial T2 over many segments or using only one septal ROI does not take into account the often focal nature of myocarditis. Small edematous changes that can be distributed diffusely or focally throughout the whole myocardium, thereby involving only small parts of many different segments may easily be overlooked when “averaging” T2 values.

Thavendiranathan et al. already noted a wider distribution of T2 values in edematous regions and discussed that this may reflect heterogeneity in tissue changes, just as there is an epicardial predominance of injury by LGE [7]. In the present study, we aimed to account for such an increased inhomogeneity in myocardial water content by elaborating on the potential of parameters like pixel-SD and madSD as novel diagnostic criteria in the diagnosis of ACM. The good diagnostic performance achieved by a combination of madSD and maxT2 favors our hypothesis that the combination of these parameters adequately reflects the different manifestations of myocardial edema in ACM, i.e. i) a focus that is confined to one or a couple of adjacent segments resulting in an increased segmental T2 and increased inhomogeneity, ii) a focus that is subdivided among several segments, leading to increased inhomogeneity but normal overall segmental and global T2 values, and iii) a diffuse edematous process that may be detected by either of the two parameters maxT2 and madSD. Our data for the present patient cohort indicate, however, that maxT2 might play only a minor, although not negligible role for the detection of myocardial edema: in sequential tests of logistic regression models, madSD showed a significant effect when maxT2 was already accounted for; conversely, maxT2 did not show an additional significant effect when added to a model that did already contain the predictor madSD. Moreover, ROC analysis showed that classification according to madSD always resulted in higher or equal true positive rates than classifications according to maxT2. The importance of both parameters as well as the defined cut-offs therefore need further validation in a larger study cohort.

In addition, further studies should look to what extent madSD is influenced by the sequence type and field strength. Applying madSD and maxT2 in a larger patient cohort at 3 T and using different sequence types therefore would be of great interest to future research.

The clinical implications of abnormal myocardial T2 still remain unclear. Nevertheless, they are believed to reflect reversible pathology [37]. In patients with clinically suspected acute myocarditis, myocardial edema has been associated with LV function recovery, indicating that the observed increase of EF may be due to the recovery of reversibly injured (edematous) myocardium [38]. Further studies should investigate this association of edematous myocardium with functional recovery as well as the potential prognostic implications of edema imaging.

We observed a certain association of the presence of myocardial edema defined by the novel diagnostic criteria with the serum levels of different cardiac biomarkers. Similar observations had also been reported previously in a conference abstract [39]. Whether myocardial T2 alterations are directly related to the biochemical severity of myocarditis, as suggested by our results, remains to be confirmed in future studies.

Only 23 out of 31 ACM patients demonstrated visual edema on T2 BB images, although none of the HV showed false-positive signal alterations in a pure visual analysis. Performing a semi-quantitative analysis as it is recommended in the LL criteria by calculating the T2-ratio [6, 22] resulted in two more cases diagnosed with edema (25 out of 31), but also in 10 out of 30 HV showing a false-positive T2-ratio >1.9. These observations underline that both, qualitative and semi-quantitative T2-weighted imaging are limited by moderate sensitivities and/or specificities and should be replaced in the future by more reliable quantitative techniques such as T2-mapping.

### Study limitations

The results shown here are explorative and need further confirmation, re-estimation of model coefficients or cut-off values as well as validation in future studies with larger patient cohorts.

We did not perform EMB in our patient cohort, but decided to use a CMR-based approach in consideration of the LL criteria [6] for confirmation of the clinical diagnosis “ACM”. Therefore, CMR with LL criteria served as our reference method, what represents a realistic approach for clinical routine in the setting of ACM, where EMB is limited by its invasiveness and by the possible associated inherent procedural risks and therefore performed only in the minority of cases with specific indications [40]. Therefore, our study design takes into account the more and more prominent role of CMR in the diagnostic work-up of ACM that potentially may lead towards a redefinition of the diagnostic gold standard in the future [41] when including more non-invasive imaging criteria to confirm the diagnosis.

Our approach using CMR with LL criteria as a reference leads to another study limitation: as the presence of two out of three LL criteria was the criterion for patients to be included in the study, we were not able to compare the diagnostic potential of T2-mapping to LL criteria, as those had an artificial sensitivity and specificity of nearly 100 %. This should be completed in a further validation study investigating the additional diagnostic value of T2-mapping to LL criteria.

## Conclusions

Establishing a timely and correct diagnosis in the setting of acute myocarditis remains a challenging task and any improvements in diagnostic performance and confidence are highly desirable. Previous attempts to detect and objectify myocardial edema as one of the hallmarks of acute inflammation by means of myocardial T2 mapping have given mixed results also because of a large overlap of absolute T2 values in disease and health. In our study, we suggest a novel approach to the quantitative analysis of T2 maps. By means of a set of parameters that better account for the inhomogeneity of disease manifestation in acute myocarditis we were able to define cut-off values for derived T2 measures (maxT2 and madSD) that, if combined, exhibit high diagnostic sensitivity and specificity in patients with CMR-proven ACM. If confirmed by future studies, this set of parameters hold the potential to overcome the hurdles of T2-mapping and promise to make it into a valuable tool in the routine diagnostic work-up for ACM.

## Abbreviations

ACM: acute myocarditis
AIC: Akaike information criterion
AUC: area under the curve
CAD: coronary artery disease
CK: creatine kinase
CMR: cardiovascular magnetic resonance
DCM: dilative cardiomyopathy
EGE: early gadolinium enhancement
EGEr: early gadolinium enhancement ratio
EMB: endomyocardial biopsy
GraSE: Gradient Spin Echo sequence
HV: healthy volunteers
IQR: inter-quartile-range
LGE: late gadolinium enhancement
LL criteria: Lake Louise consensus criteria
LV: left ventricle
MAD: mean absolute deviation
MESE: multi echo spin echo sequence
NT-proBNP: brain natriuretic peptide
ROC: receiver operating curve
ROI: region of interest
SAX: short axis
SD: standard deviation
SSFP: steady-state free-precession
T: Tesla
T2 BB: T2 black blood
TnT: troponin T
